# Mechanistic Principles of Exciton–Polariton Relaxation

**DOI:** 10.1021/acs.jpclett.6c00405

**Published:** 2026-04-03

**Authors:** Ian Haines, Arshath Manjalingal, Logan Blackham, Saeed Rahmanian Koshkaki, Arkajit Mandal

**Affiliations:** † Department of Chemistry, 14736 Texas A&M University, College Station, Texas 77843, United States

## Abstract

Exciton-polaritons are light–matter hybrid quasi-particles that have emerged as a flexible platform for developing quantum technologies and engineering material properties. However, the fundamental mechanistic principles that govern their dynamics and relaxation remain elusive. In this work, we provide the microscopic mechanistic understanding of the exciton-polariton relaxation process that follows from an excitation in the upper polariton. Using both mixed quantum-classical simulations and analytical analysis, we reveal that phonon-induced upper-to-lower polariton relaxation proceeds via two steps: the first step is a vertical interband transition from the upper to the lower polariton, which is followed by a second step that is a phonon-induced Fröhlich scattering within the lower polariton. We find that in materials of finite thickness (which include filled cavities), phonon-induced polaritonic intraband Fröhlich scattering is significantly suppressed. We show that the microscopic origin of this suppression is phonon-fluctuations synchronization (or self-averaging) due to the polaritonic spatial delocalization in the quantization direction. Finally, we show that the same phonon fluctuation-synchronization effect plays a central role across polaritonic relaxation pathways, and we derive simple analytical expressions that relate a material’s finite thickness to the corresponding relaxation rate constants.

## Graphical abstract

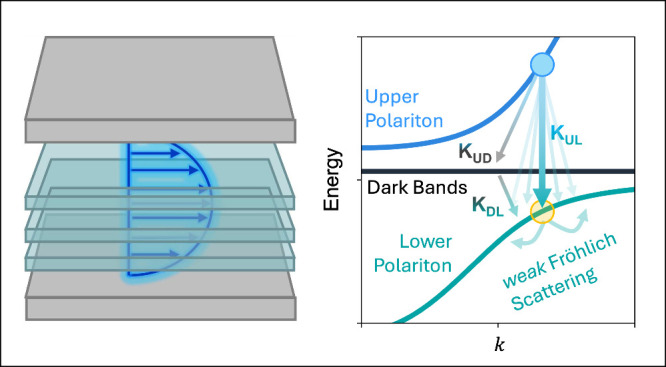

Exciton-polaritons, formed by the strong coupling of materials or organic molecules to quantized radiation inside an optical cavity,^1–6^ are an emerging platform that shows growing promise for application in classical computing,^7–10^ neuromorphic computing,^11–13^ quantum computing,^14–16^ chemical catalysis,^17–26^ and information transduction.^27,28^ Despite extensive theoretical efforts to understand exciton–polaritons,^29–40^ the microscopic mechanisms governing their dynamics remain poorly understood. A key limitation of many theoretical studies is the reliance on simplifying approximations that contradict experimental reality. Such approximations include the long-wavelength approximation, single-emitter approximation, single-layer approximation, and single cavity-mode approximation. Although these approximations may reproduce certain spectroscopic features, they often fail to capture key dynamical aspects of exciton–polaritons, leading to ambiguities or inaccurate descriptions of exciton–polariton dynamics.

Existing theoretical work^31,37,41–43^ employs the Holstein-Tavis-Cummings model (or its multimode generalization) to study the exciton-polariton relaxation and utilizes a simple 1D-chain model, effectively representing a single layer, (depicted in Figure 1a) to describe the excitonic subsystem. In contrast, most experiments^30,44–49^ use a filled cavity or partially filled cavities with multilayered materials, (as illustrated in Figure 1b) whose evolution is starkly different, and plays a crucial role in polariton relaxation dynamics. Relative to a single-layer system, a filled cavity houses additional dark excitonic manifolds^45,50,51^ that open new relaxation pathways (illustrated in Figure 1) from the upper polariton to the lower polariton. Presently, a microscopic understanding of this polariton relaxation process, especially in filled Fabry-Pérot optical cavities, is missing. In this work, using mixed quantum-classical dynamics, namely the multitrajectory Ehrenfest approach,^3,42,52–54^ as well as an analytical analysis, we provide a microscopic understanding of the polariton relaxation process following an initial excitation in the upper polariton branch of a multilayer material inside an optical cavity.

**Figure 1:**
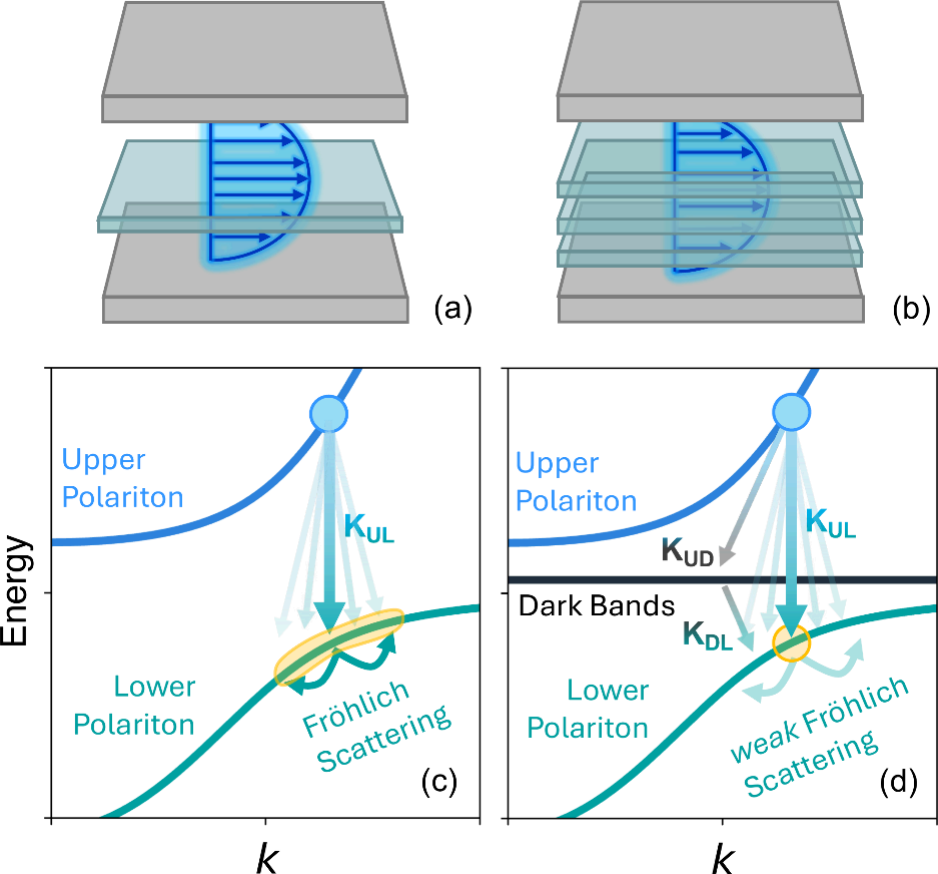
Schematic of polariton dynamics. (a and b) Schematic drawing of a single-layer and multilayered material in an optical cavity. (c and d) Schematic drawing of the single-layer and multilayer band structures with energy on the *y*-axis and wavevector (*k*) along the horizontal axis. *K*_UL_ (*K*_DL_, *K*_UD_) is the relaxation rate from the upper polariton (dark states, upper polariton) to lower polariton (lower polariton, dark states).

We show that phonon-induced upper-to-lower polariton relaxation proceeds via two steps. We find that the first step is a phonon-induced interband *vertical* transition, i.e., a direct population transfer from the upper polariton to the lower polariton with **negligible** change in the in-plane polaritonic momentum. The second step is a phonon-induced intraband Fröhlich scattering within the lower polariton band. We find that this second step is significantly suppressed in a multilayered (or filled-cavity) geometry due to a *phonon-fluctuation synchronization effect*, whereby fluctuations are effectively averaged across layers due to the delocalized nature of the polaritons (via self-averaging). Note that, the layer-to-layer phonon coordinates are not synchronized (they are uncorrelated), and that because they are uncorrelated, the delocalized polaritonic coupling samples a layer-averaged coordinate whose variance is reduced. As a consequence of this, the lower polariton population remains energetically localized (i.e., *stuck*) for hundreds of femtoseconds. From another perspective, the increase in the number of dark bands appears to suppress phonon-induced Fröhlich scattering, protecting the lower polariton from appreciable Fröhlich scattering. We show that the same phonon fluctuation self-averaging plays a central role in polariton relaxation rates, which allows us to obtain simple analytical expressions connecting the number of layers of the material to various relaxation rate constants. Overall, in this work we provide a unified theoretical understanding of exciton-polariton relaxation processes.

## Model

To study the relaxation process in a filled cavity, we considered the following light–matter Hamiltonian beyond the long-wavelength approximation,^45,54,55^

1 $${Ĥ}_{LM}={Ĥ}_{X}+{Ĥ}_{b}+{Ĥ}_{c}+{Ĥ}_{bX}+{Ĥ}_{cX}$$

Here ${Ĥ}_{X}$ , ${Ĥ}_{b}$ and ${Ĥ}_{c}$ describe the bare exciton, phonon and cavity Hamiltonians. ${Ĥ}_{bX}$ and ${Ĥ}_{cX}$ describe the interaction between exciton and phonons as well as exciton and the cavity photons, respectively. The excitonic part of the light–matter Hamiltonian is written as

2 $${Ĥ}_{X}=\overset{N,M}{\underset{n,m}{\sum }}\epsilon_{0}{\mathrm{X̂}}_{n,m}^{†}{\mathrm{X̂}}_{n,m}-\tau \left({\mathrm{X̂}}_{n+1,m}^{†}{\mathrm{X̂}}_{n,m}+\mathrm{h.c.}\right)=\overset{M}{\underset{m,k}{\sum }}\epsilon_{k}{\mathrm{X̂}}_{k,m}^{†}{\mathrm{X̂}}_{k,m}$$

where ${\mathrm{X̂}}_{n,m}^{†}$ and ${\mathrm{X̂}}_{n,m}$ are excitonic creation and annihilation operators at site *n* and layer *m*, ϵ_0_ is the on-site energy, and τ is the intralayer nearest-neighbor hopping integral. Further, ${\mathrm{X̂}}_{k,m}^{†}=\frac{1}{\sqrt{N}}\underset{n}{\sum }e^{ik\cdot x_{n}}{\mathrm{X̂}}_{n,m}^{†}$ is the partial Fourier transformed excitonic operator with the in-plane wavevector (momentum) *k* and with the associated band energy ϵ_*k*_ = [ϵ_0_ – 2τ cos­(*kα*)] (which results from the intralayer nearest neighbor hopping term τ) where α is the lattice constant. Note, ${\mathrm{X̂}}_{k,m}^{†}$ creates a single excitation (an exciton) of energy ϵ_*k*_ which is delocalized within the *m*th layer. The bare phonon Hamiltonian is written as a collection of harmonic operators of the form

3 $${Ĥ}_{b}=\overset{N}{\underset{n}{\sum }}\overset{M}{\underset{m}{\sum }}\left(\frac{{\mathrm{p̂}}_{n,m}^{2}}{2}+\frac{1}{2}\omega^{2}{\mathrm{q̂}}_{n,m}^{2}\right)$$

where ${\mathrm{p̂}}_{n,m}$ and ${\mathrm{q̂}}_{n,m}$ are the momentum and position operators of the phonons and ω is the phonon frequency. The exciton–phonon coupling term is written as

4 $${Ĥ}_{bX}=\gamma \overset{N}{\underset{n}{\sum }}\overset{M}{\underset{m}{\sum }}{\mathrm{q̂}}_{n,m}{\mathrm{X̂}}_{n,m}^{†}{\mathrm{X̂}}_{n,m}=\frac{\gamma }{N}\underset{k,k^{\prime }}{\sum }\overset{M}{\underset{m}{\sum }}{\mathrm{X̂}}_{k,m}^{†}{\mathrm{X̂}}_{k^{\prime },m}\underset{n}{\sum }{\mathrm{q̂}}_{n,m}e^{i\left(k-k^{\prime }\right)x_{n}}$$

where γ is the exciton–phonon coupling strength. Note we obtain the phase factor $e^{i\left(k-k^{\prime }\right)x_{n}}$ in the second line of eq 4 by writing ${\mathrm{X̂}}_{n,m}^{†}$ in reciprocal space while keeping the phonon ${\mathrm{q̂}}_{n,m}$ in the real space. The cavity part of the light–matter Hamiltonian considers a set of confined radiation modes (with differing in-plane momentum) in a Fabry-Pérot optical cavity and is written as (using *ℏ* = 1 a.u.)

5 $${Ĥ}_{c}=\underset{k}{\sum }\omega_{k}{â}_{k}^{†}{â}_{k}$$

where ${â}_{k}^{†}$ and ${â}_{k}$ are creation and annihilation operators for wavevector *k*. The photon frequency is described by

6 $$\omega_{k}=\frac{c}{\eta }\left|k\mathrm{x⃗}+k_{0}\mathrm{y⃗}\right|$$

where *c* is the speed of light, η is the refractive index, and x⃗ and y⃗ are unit vectors along *x* and *y* directions. Further, the photon wavevector in the quantization direction (*y*-direction) is $k_{0}=\frac{\pi }{L}$ , where L=1000⁡Å is the distance between the two cavity mirrors. The in-plane momentum is $k=\frac{2\pi n_{x}}{N\cdot \alpha }$ where we have imposed a periodic boundary condition in the lateral direction with *n*_*x*_ = 0, ± 1, ± 2, and α = 12 Å. Finally, we consider the exciton-cavity interaction ${Ĥ}_{cX}$ beyond the long-wavelength approximation which is written as

7 $${Ĥ}_{cX}=\underset{n,m,k}{\sum }\frac{\Omega_{k}}{\sqrt{N}}\left({\mathrm{X̂}}_{n,m}^{†}{â}_{k}e^{ikx_{n}}+\mathrm{h.c.}\right)\sin\left(k_{0}\cdot y_{m}\right)$$

where $\Omega_{k}=\sqrt{\frac{\omega_{0}}{\omega_{k}}}\Omega_{0}$ is the light–matter coupling strength and *y_m_= mα_y_* with *α_y_* as the interlayer spacing. In this work, the exciton-photon subsystem is treated quantum mechanically while the phonon degrees of freedoms are evolved quasi-classically using the multitrajectory Ehrenfest approach. We restrict our dynamics to the single excitation subspace such that the wave function describing the exciton-photon subspace is written as

8 $$\left|\Psi \left(t\right)\right\rangle =\underset{k}{\sum }c_{k}\left(t\right)\left|1_{k}\right\rangle +\underset{n,m}{\sum }b_{n,m}\left(t\right)\left|n,m\right\rangle$$

with *c*_*k*_(*t*) and *b*_*n*,*m*_(*t*) are the time-dependent coefficients, $\left|1_{k}\right\rangle \equiv {â}_{k}^{†}\left|\mathrm{0̅}\right\rangle$ and $\left|n,m\right\rangle \equiv {\mathrm{X̂}}_{n,m}^{†}\left|\mathrm{0̅}\right\rangle$ with |0̅⟩ representing the light–matter ground state with no photons in the cavity nor excitations in the material (i.e., material in its ground state).

The bare exciton-polariton Hamiltonian ${Ĥ}_{EP}$ = ${Ĥ}_{LM}-{Ĥ}_{b}-{Ĥ}_{bX}$ can be re-expressed as

9 $${Ĥ}_{EP}=\underset{k,i\in \pm }{\sum }\omega_{k,i}{\mathrm{P̂}}_{k,i}^{†}{\mathrm{P̂}}_{k,i}+\underset{k,d}{\sum }\epsilon_{k}{\mathrm{X̂}}_{k,d}^{†}{\mathrm{X̂}}_{k,d}$$

where $\left\{{\mathrm{P̂}}_{k,\pm }^{†}\right\}$ are the upper (+) and lower (−) polariton operators and $\left\{{\mathrm{X̂}}_{k,d}^{†}\right\}$ are dark excitonic operators. The polaritonic operators are defined as

10 $${\mathrm{P̂}}_{k,+}^{†}=\sin\left(\theta_{k}\right){â}_{k}^{†}+\cos\left(\theta_{k}\right){\mathrm{X̂}}_{k,B}^{†}$$

11 $${\mathrm{P̂}}_{k,-}^{†}=\cos\left(\theta_{k}\right){â}_{k}^{†}-\sin\left(\theta_{k}\right){\mathrm{X̂}}_{k,B}^{†}$$

where ${\mathrm{X̂}}_{k,B}^{†}=\frac{1}{\sqrt{S}}\overset{N_{L}}{\underset{m}{\sum }}\sin\left(k_{0}\cdot y_{m}\right){\mathrm{X̂}}_{k,m}^{†}$ is the bright exciton creation operator that creates an exciton delocalized in all *N*_*L*_ layers with in-plane momentum *k* and θ_*k*_ = $\frac{1}{2}\tan^{-1}\left[2\sqrt{S}\Omega_{k}/\left(\omega_{k}-\epsilon_{k}\right)\right]$ is the mixing angle with *S* = *∑*_*m*_ sin^2^(*k*_0_ · *y*_*m*_). Further, ${\mathrm{X̂}}_{k,d}^{†}=\underset{m}{\sum }D_{m,d}{\mathrm{X̂}}_{k,m}^{†}$ where $D_{m,d}$ are orthonormal coefficients $\left(\underset{m,m^{\prime }}{\sum }D_{m,d}D_{m^{\prime },d^{\prime }}=\delta_{m,m^{\prime }}\delta_{d,d^{\prime }}\right)$ which satisfy $\underset{m}{\sum }D_{m,d}\cdot \sin\left(k_{0}\cdot y_{m}\right)=0$ , for all *d*, such that $\left[{\mathrm{X̂}}_{k,d}^{†},{\mathrm{X̂}}_{k,B}\right]=0$ .

## Initialization

We initialize the exciton-polariton wave function as a linear combination of upper-polariton states

12 $$\left|\Psi \left(0\right)\right\rangle =\underset{k\in E_{init}}{\sum }\alpha_{k}{\mathrm{P̂}}_{k,+}^{†}\left|\mathrm{0̅}\right\rangle$$

where $E_{init}$ defines a subspace corresponding to the lower polariton states of energy ω_*k*,+_ that lie within an energy window *E*_init_ – Δ/2 < ω_*k*,–_ < *E*_init_ + Δ/2. Here, the lower polariton operator ${\mathrm{P̂}}_{k,-}^{†}$ is obtained by diagonalizing the exciton-polariton Hamiltonian (in the absence of the phonons). We select the coefficients α_*k*_ by minimizing

13 $$\underset{n}{\sum }|\left\langle \mathrm{0̅}\left|{\mathrm{X̂}}_{n}\right|\Psi \left(0\right)\right\rangle {|}^{2}\left(n-N/2\right)$$

such that |Ψ(0)⟩ represents an energetically as well as a spatially localized wave function at *t* = 0. And finally, the initial phonon positions and momentum {*q*_*n*,*m*_(0), *p*_*n*,*m*_(0)} are sampled from the Wigner distribution ${\left[{\mathrm{\rho ̂}}_{\mathrm{phn}}\right]}_{W}\propto e^{-2\tanh\left(\beta \omega \right){Ĥ}_{b}/\omega }$ .

## Quantum Dynamical Approach

Due to the formidable nature of exact quantum dynamical simulations, here, we use the multitrajectory Ehrenfest approach (as in recent works^29–32,35,42,52,56^ ) to propagate the quantum dynamics of the light–matter system. In this mixed quantum-classical approach, the phonons are propagated quasi-classically using the following equations of motion:

14 $${ṗ}_{n,m}\left(t\right)=-\left\langle \Psi \left(t\right)\right|\frac{\partial {Ĥ}_{LM}}{\partial q_{n,m}}\left|\Psi \left(t\right)\right\rangle ,{\mathrm{q̇}}_{n,m}\left(t\right)=p_{n,m}\left(t\right)$$

We integrate these equations of motion and update our phonons via the velocity-Verlet algorithm.^57^ The exciton-polariton part is propagated quantum mechanically using an efficient split-operator approach combined with a bright-layer unitary transformation.^54^ Short-time propagation of the exciton-polariton wave function over a time step *δt* is written as

15 $$\left|\Psi \left(t+\delta t\right)\right\rangle ={Û}_{pol}e^{-i{Ĥ}_{EP}\delta t}{Û}_{pol}^{†}e^{-i{Ĥ}_{env}\delta t}\left|\Psi \left(t\right)\right\rangle$$

where ${Û}_{pol}^{†}$ denotes the unitary transformation that maps the real-reciprocal space basis to the polaritonic basis, and ${Ĥ}_{env}={Ĥ}_{b}+{Ĥ}_{bX}$ , which represents the phononic contribution to the Hamiltonian. Furthermore, both ${Ĥ}_{EP}$ and ${Ĥ}_{env}$ are each diagonal in their own natural bases; their combination can be evaluated through an element-wise operation rather than a full matrix multiplication, leading to a substantial reduction in computational cost. That is, in our split operator approach, we use the real-space description for ${Ĥ}_{env}$ , with ${Ĥ}_{bX}=\gamma \underset{n,m}{\sum }q_{n,m}\left(t\right){\mathrm{X̂}}_{n,m}^{†}{\mathrm{X̂}}_{n,m}$ which is a function of the classical variable *q*_*n*,*m*_(*t*), while we use reciprocal representation for ${Ĥ}_{EP}$ . Our full mixed quantum-classical dynamics method is described in ref 54.

## Parameters

For the simulations presented below, our parameters are closely related to perovskite material,^58^ although our result are generic for exciton-polaritons. The on-site excitonic energy is set to ϵ_0_ = 3.2 eV, and the nearest-neighbor hopping parameter is chosen as τ = 400 cm^–1^, using 40000 sites per layer. The intralayer lattice constant is set to α=12⁡Å , and an interlayer spacing is set to $\alpha_{y}=40Å$ . The two mirrors are separated by a distance L=1000⁡Å , and light–matter coupling is Ω_0_ = 241.7 meV for a single-layer material. For multilayered materials we renormalize Ω_0_ by $1/\sqrt{S}$ to make a fair comparison wherein the overall Rabi splitting is the same regardless of the thickness of the material. The phonon frequency is ω = 720 cm^–1^ and the exciton–phonon coupling strength is γ = 3.76 × 10^–4^ a.u.

## Extracting Rate Constants

The relaxation dynamics among the upper, lower, and dark polaritonic states can be modeled as a three-state kinetic model (coupled rate equations) involving a set of rate constants which is written as

16 $$\frac{d}{dt}\mathrm{P⃗}=K\mathrm{P⃗}$$

where $\mathrm{P⃗}={\left[P_{+}\left(t\right),P_{d}\left(t\right),P_{-}\left(t\right)\right]}^{T}$ is the population vector, which includes the upper polariton, dark excitons, and lower polariton populations, respectively, where *T* denotes a matrix transpose. These quantities are computed as

17 $$P_{+}\left(t\right)=\underset{k}{\sum }\left\langle \mathrm{0̅}\right|{\mathrm{P̂}}_{k,+}\left|\psi \left(t\right)\right\rangle$$

18 $$P_{-}\left(t\right)=\underset{k}{\sum }\left\langle \mathrm{0̅}\right|{\mathrm{P̂}}_{k,-}\left|\psi \left(t\right)\right\rangle$$

19 $$P_{d}\left(t\right)=1-P_{+}\left(t\right)-P_{-}\left(t\right)$$

The rate constant matrix **K** describing the interconversion among these states is expressed as

20 $$K=\left[\begin{array}{ccc} -k_{UL}-k_{UD} & k_{DU} & k_{LU}\\ k_{UD} & -k_{DL}-k_{DU} & k_{LD}\\ k_{UL} & k_{DL} & -k_{LU}-k_{LD} \end{array}\right]$$

where each *k*_*ij*_ denotes the transition rate constant from state *i* to *j* (e.g., *k*_*UL*_: upper → lower, *k*_*UD*_: upper → dark, *k*_*LD*_: lower → dark), as schematically illustrated in Figure 1. This system of coupled first-order linear differential equations can be analytically solved via eigenvalue decomposition, yielding the expression given as

21 $$\mathrm{P⃗}\left(t\right)=e^{Kt}\mathrm{P⃗}\left(0\right)$$

The rate constants are extracted by fitting the analytical model to the computed population dynamics. We note that the MFE approach adapted here is known to break detail balance at longer times. However, recent work on cavity modified electron transfer kinetics^59–61^ illustrate that this rate fitting scheme reproduces the predictions of Marcus theory to reasonable accuracy.

Figure 2 presents the relaxation dynamics in single-layer material and in a multilayered material (or material of a finite thickness) inside an optical cavity. Here the system is initially excited within an energy window of 3.5 ± 0.2 eV in the upper polariton. The initial polariton-band-resolved population is illustrated in Figure 2a and Figure 2e for the single-layer and five-layer configurations, respectively.

**Figure 2:**
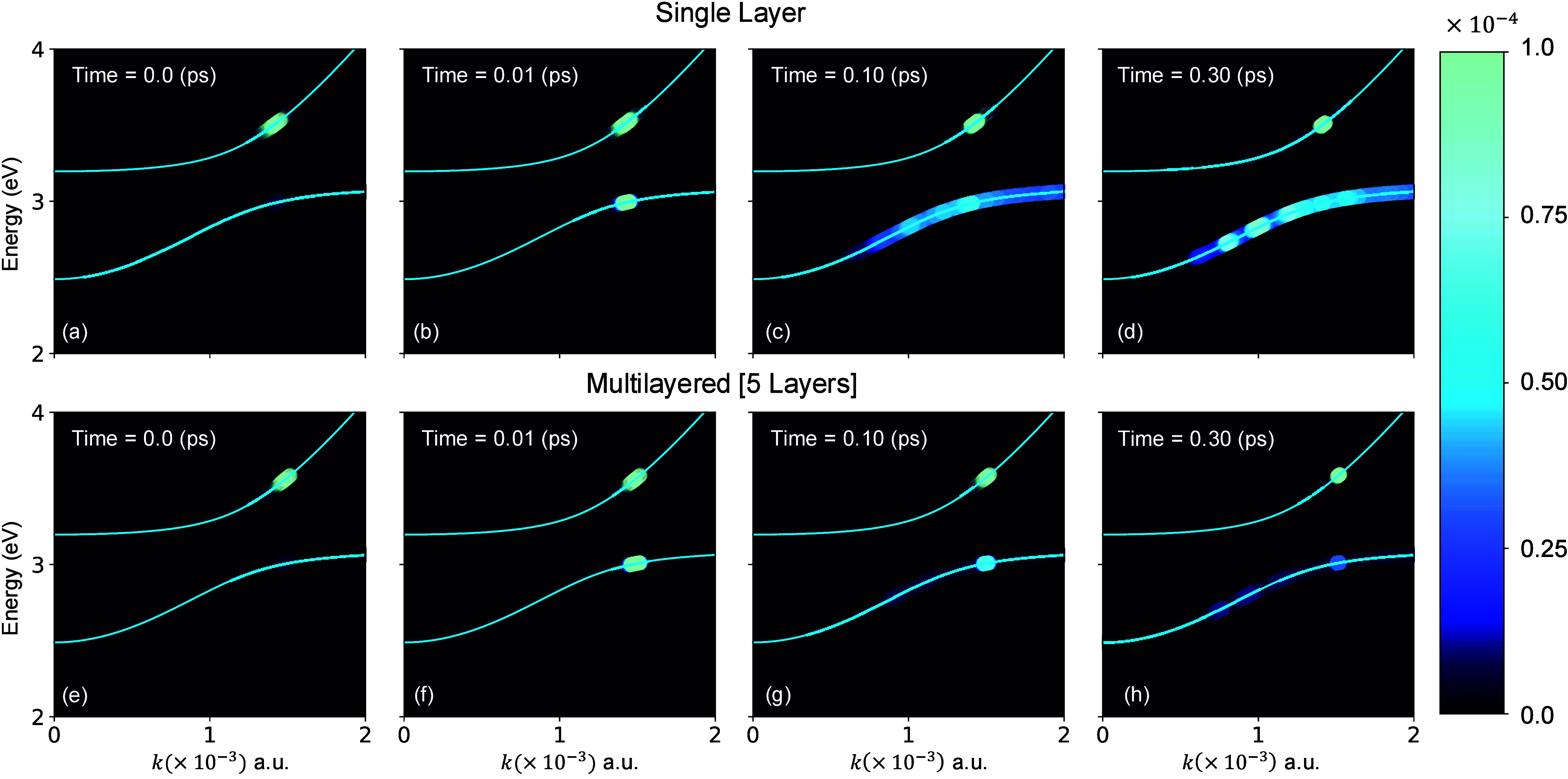
Polariton-band-resolved relaxation dynamics. Polariton-band-resolved population relaxation dynamics in a single-layer material (a–d) and in multilayered material (5 layers) (e–h), following an excitation to the upper polariton centered at 3.5 eV. The plots from left to right display a snapshot at the initial time, 0.01, 0.10, and 0.30 ps of the population (blue circles), where there is not only a transfer of population from the upper to lower polariton, but also Fröhlich scattering the lower polariton. The degree of this scattering (a–d) is much greater for a single layer of material than in the multilayered material (e–h), owing to the “phonon-fluctuation synchronization effect” in the multilayered case.

Figure 2b, and f shows the lower polariton branch population after propagation for 0.01 ps, and this relaxation process can be seen to approximately preserve polaritonic momentum leading to a vertical (*k* → *k*) transition. While a phonon-induced relaxation that enables fast population transfer between polaritonic branches is expected, the vertical nature of the transition is counterintuitive. In the following (Figure 3) we provide a microscopic mechanistic understanding of this vertical nature of the polariton relaxation.

**Figure 3:**
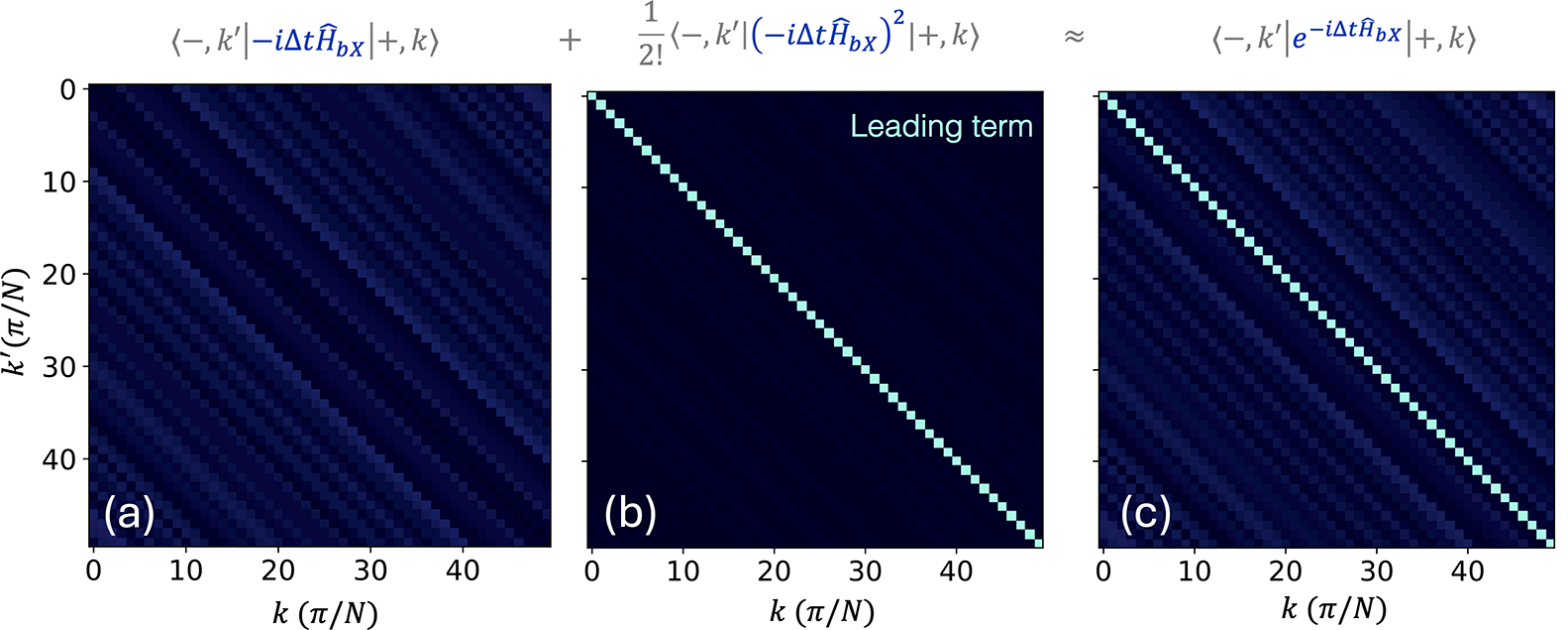
The vertical nature of the polariton relaxation. (a–c) Upper to lower polariton transfer matrix elements for the first-order (a), second-order (b), and full exponential propagator (c). Note that here we are presenting the absolute values of the matrix elements. Here, we consider a system comprising 40,000 sites and a time step of Δ*t* = 50 a.u.

Figure 2c-d shows that, at longer times, the population within the lower polariton for a single-layered material is highly scattered owing to the phonon fluctuations. Interestingly, in the multilayered material, this phonon induced scattering is significantly suppressed, leading to a relaxation process that continues to conserve momentum for a longer duration (see Figure 2g-h), which we attribute to a phonon-fluctuation synchronization effect that reduces the magnitude of the phonon fluctuations, suppressing the Fröhlich scattering.

Our numerical results demonstrate that the first step of the polaritonic relaxation is (polariton) momentum conserving. This is surprising, as the phonon-induced relaxation that originates from ${Ĥ}_{bX}$ does not directly show an in-plane momentum conservation, as a transition from *k* → *k*′ appear to be allowed. To clearly see this, consider the second line of eq 4 which can be expanded (replace $\left\{{\mathrm{X̂}}_{n,m}^{†}\right\}$ with $\left\{{\mathrm{P̂}}_{k,i}^{†},{\mathrm{X̂}}_{k,d}^{†}\right\}$ ) to obtain the following polariton-phonon coupling term responsible for the upper-to-lower polariton relaxation:

22 $$P_{-}{Ĥ}_{bX}P_{+}=\gamma \underset{k,k^{\prime }}{\sum }{\mathrm{P̂}}_{k,-}^{†}{\mathrm{P̂}}_{k^{\prime },+}\left[\cos\left(\theta_{k}\right)\sin\left(\theta_{k^{\prime }}\right)Q\left(t\right)\right]$$

Here, $P_{\pm }=\underset{k}{\sum }{\mathrm{P̂}}_{k,\pm }^{†}{\mathrm{P̂}}_{k,\pm }$ are projection operators and $Q\left(t\right)=\underset{n}{\sum }e^{-i\left(k-k^{\prime }\right)x_{n}}\frac{1}{S}\underset{m}{\sum }q_{n,m}\left(t\right)\sin^{2}\left(k_{0}\cdot y_{m}\right)$ is a time-dependent variable that encodes the dynamical fluctuations of the phonons. Clearly eq 22 suggests that ${\mathrm{P̂}}_{k,+}^{†}\to {\mathrm{P̂}}_{k^{\prime },+}$ transitions are allowed for *k* ≠ *k*′ which contradicts the results presented in Figure 2a-b and Figure 2e-f. Below we resolve this apparent contradiction.

## Polaritonic Vertical Transition

To find the microscopic mechanism of the (predominantly) vertical nature of the polaritonic transition at early times, we compute the transition probability to the lower polariton, following an excitation to the upper polariton, after a short time propagation of Δ*t*, which is computed as

$$P_{-,k^{\prime }}\left(\Delta t\right)\approx {\left|\left\langle -,k^{\prime }\right|e^{-i{Ĥ}_{bX}\Delta t}e^{-i{Ĥ}_{EP}\Delta t}\left|+,k\right\rangle \right|}^{2}={\left|\left\langle -,k^{\prime }\right|e^{-i{Ĥ}_{bX}\Delta t}\left|+,k\right\rangle \right|}^{2}$$ 23

where $\left|+,k\right\rangle ={\mathrm{P̂}}_{k,+}^{†}\left|\mathrm{0̅}\right\rangle$ and $\left|-,k\right\rangle ={\mathrm{P̂}}_{k,-}^{†}\left|\mathrm{0̅}\right\rangle$ . Note that we arrive at the second line by using the fact that $e^{-i{Ĥ}_{EP}\Delta t}\left|+,k\right\rangle =e^{-iE_{+,k}\Delta t}\left|+,k\right\rangle$ since the polaritonic states are the eigenstates of the bare exciton-polariton Hamiltonian ${Ĥ}_{0}$ . Figure 3c presents $\left|\left\langle -,k^{\prime }\left|e^{-i{Ĥ}_{bX}\Delta t}\right|+,k\right\rangle \right|$ , which clearly demonstrates that the diagonal transition elements dominate, leading to vertical transitions. To investigate the origin of this behavior, we consider the Taylor expansion of $e^{-i{Ĥ}_{bX}\Delta t}$ , written as
24

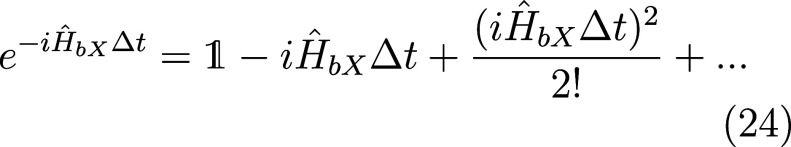

The term 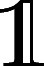 will not contribute to the upper-to-lower polariton transition and can be dropped. Interestingly, we find that the contribution of the term linear in Δ*t* is also vanishingly small (see Figure 3a) while the leading non-negligible contribution comes from the second order term in Δ*t* (see Figure 3b) with its diagonal elements dominating.

This behavior can be analytically understood by noting that ${\left(\underset{n}{\sum }{\mathrm{X̂}}_{n}^{†}{\mathrm{X̂}}_{n}R_{n}\right)}^{p}=\underset{n}{\sum }{\mathrm{X̂}}_{n}^{†}{\mathrm{X̂}}_{n}R_{n}^{p}$ , where *p* ∈ {1, 2, ..., *N*}, allowing for the transition element to be expressed as

25 $$\langle -,k^{\prime }\left|e^{-i{Ĥ}_{bX}\Delta t}\right|+,k\rangle =C_{kk^{\prime }}\underset{n}{\sum }\frac{e^{i\left(k^{\prime }-k\right)\cdot x_{n}}}{N}\left(1-e^{-i\gamma q_{n,m}\Delta t}\right)\approx C_{kk^{\prime }}\left\langle 1-e^{-i\gamma q\Delta t}\right\rangle \underset{n}{\sum }\frac{e^{i\left(k^{\prime }-k\right)\cdot x_{n}}}{N}=C_{kk^{\prime }}\left\langle 1-e^{-i\gamma q\Delta t}\right\rangle \cdot \delta_{kk^{\prime }}$$

where $C_{kk^{\prime }}=\underset{m}{\sum }\sin\theta_{k^{\prime }}\cos\theta_{k}\frac{\sin^{2}\left(k_{0}\cdot y_{m}\right)}{S}$ and ⟨···⟩ represents phase space averaging. Importantly, here we arrive at the third line by utilizing the fact that for the relevant dynamics, *k* – *k*′ ≪ 1/α (i.e., using separation of length-scales) which is valid for α=12⁡Å set here and in typical materials. Using the fact that typical relaxation process occur between the anticrossing points, the following criteria can be derived

26 $$\frac{\eta }{c}\sqrt{\epsilon_{0}^{2}-\omega_{0}^{2}}\ll \frac{1}{\alpha }$$

for which a material will exhibit vertical transition. It is worth noting that artificially scaling lattice constant (as is done in recent work^30,33,43,62^ ) will miss the vertical nature of this polaritonic relaxation as such model will not satisfy the criteria in eq 26. Note that the magnitude of the transition is governed by the phonon disorder term

27 $$\left\langle 1-e^{-i\gamma q\Delta t}\right\rangle =i\gamma \Delta t\left\langle q\right\rangle +\frac{{\left(\gamma \Delta t\right)}^{2}}{2}\left\langle q^{2}\right\rangle +\cdot \cdot \cdot$$

Here, the first-order term is negligible as ⟨q⟩≈0 for harmonic phonon modes. In contrast, the second-order term $\left\langle q^{2}\right\rangle ={\left[2\omega \tanh\left(\beta \omega /2\right)\right]}^{-1}\approx \frac{1}{\beta \omega^{2}}$ (with the approximated form obtained in the classical limit) is the leading (non-negligible) term which corroborates our findings in Figure 3.

As a result of this vertical transition, we observe an energetically localized density in the lower polariton at short times (see Figure 2b and Figure 2f). In the single-layer material, at longer times, this energetically localized population density spreads out due to phonon-induced Fröhlich scattering. However, in the multilayered material (or material of finite thickness), this spreading is significantly suppressed. Below we show that this is due to a phonon fluctuation synchronization effect, where phonon fluctuations are averaged over multiple layers.

## Fröhlich scattering

To clearly understand the suppression of the Fröhlich scattering in the lower-polariton, we consider the phonon interaction term projected within the bright excitonic subspace that is written as

28 $$P_{B}{Ĥ}_{bX}P_{B}=\gamma \underset{n}{\sum }{\mathrm{X̂}}_{n,B}^{†}{\mathrm{X̂}}_{n,B}{\mathrm{q̅}}_{n}$$

where ${\mathrm{q̅}}_{n}=\frac{1}{S}\underset{m}{\sum }\sin^{2}\left(k_{0}\cdot y_{m}\right)q_{n,m}\left(t\right)$ is layer-averaged phonon fluctuation and $P_{B}=\underset{n}{\sum }{\mathrm{X̂}}_{n,B}^{†}{\mathrm{X̂}}_{n,B}$ . Because the bright exciton couples only to this weighted average over layers (i.e., ${\mathrm{q̅}}_{n}$ ), local phonon fluctuations are partially canceled in the sum, as they are uncoupled and random, which leads to the effective suppression of the phonon-induced disorder. We refer to this as the phonon-fluctuation synchronization effect. In our recent work,^54^ we have shown that this phonon-fluctuation synchronization effect enables enhanced coherent transport and significantly increases coherence lifetime of the polariton. Note the arguments based on the bright layer projected phonon couplings in eq 28 are valid when the dark layers are energetically decoupled due to the light–matter couplings with upper and lower polaritons lying above and below the dark-excitonic energy. The extent of the suppression of phonon fluctuations can be quantified by the effective phonon variance,^63–65^ defined as

29 $$\left\langle {\mathrm{q̅}}^{2}\right\rangle =\frac{1}{N}\underset{n}{\sum }{\mathrm{q̅}}_{n}^{2}=\left\langle q^{2}\right\rangle \cdot \frac{1}{S^{2}}\overset{N_{L}}{\underset{m}{\sum }}\sin^{4}\left(k_{0}\cdot y_{m}\right)$$

where the factor $\frac{1}{S^{2}}\underset{m}{\sum }\sin^{4}\left(k_{0}\cdot y_{m}\right)\leq 1$ encodes the reduction due to multilayer averaging of the phonon fluctuations. The phonon fluctuation decreases with an increase in the number of layers which also increases the number of dark layers. Thus, interestingly the presence of a large number of dark layers appear to (indirectly) sequester phonon-induced fluctuation leading to suppressed Fröhlich scattering. At the same time, $\left\langle q^{2}\right\rangle \approx \frac{1}{\beta \omega^{2}}$ , such that this fluctuation synchronization can also be viewed as a suppression of the effective classical temperature which is expressed as

30 $$T_{e\mathrm{ff}}=T\cdot \frac{1}{S^{2}}\underset{m}{\sum }\sin^{4}\left(k_{0}\cdot y_{m}\right)$$

We emphasize that the suppressed *T*_eff_ does not indicate a physical reduction of temperature but rather an effective reduction of the fluctuations and dissipation that the exciton-polariton subsystem experiences.

In Figure 4, we numerically test our proposed mechanism: Fröhlich scattering is suppressed by the phonon-fluctuation synchronization effect, leading to a long-lived, *k*-space–localized population in multilayered materials. Figure 4a-b and Figure 4c-d presents the k-resolved lower polariton population (with the system initialized in the upper polariton) in a single-layer material and a multilayered material, respectively. As was shown in Figure 2, while the initial density is *k*-localized at short times in the lower polariton for both cases ( ∼10 fs), at longer times the population density remains *k*-localized only in the multilayered material. To provide a direct numerical evidence of the phonon synchronization mechanism, in Figure 4e-f we perform a quantum dynamical simulation where we sample the phonons in a constrained fashion. We set *q*_*n*,*m*_(0) = *q*_*n*,1_(0), for all layers, such that ${\mathrm{q̅}}_{n}\left(0\right)=q_{n,1}\left(0\right)$ . Under this constrained initial sampling, the weighted average of identical fluctuations over multiple layers is the same as the fluctuations themselves. Under such constrained sampling, our numerical simulation, presented in Figure 4e-f, show that the dynamics resembles the single layer scenario where the *k*-localized lower polariton density at short times (see Figure 4e) become *k*-delocalized in the same fashion (see Figure 4f) as in the single layer scenario. Note that such a scenario could be mimicked experimentally by launching coherently oscillating phonons across layers. This numerically demonstrates that the *k*-localization in the multilayered scenario originates from the phonon fluctuation averaging.

**Figure 4:**
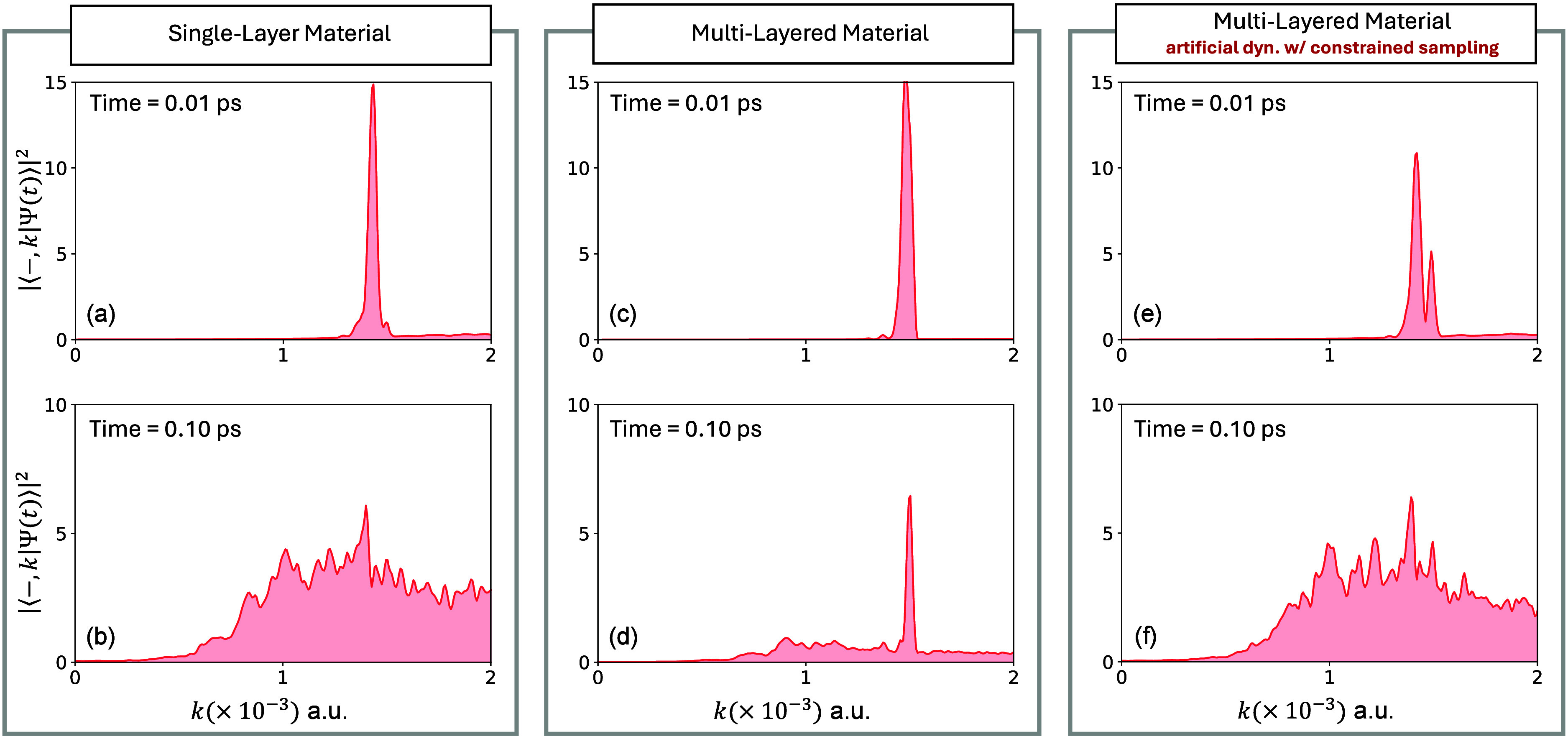
Suppression of Fröhlich scattering in multilayered materials via phonon fluctuation synchronization effect. (a–d) Relaxation dynamics in the lower polariton band in single layer vs multilayered material. (e and f) Relaxation dynamics in the lower polariton when we constrain phonon sampling. For this simulation we copied the initial position and momentum of the phonons for each layer, eliminating any difference in the phonons layer to layer.

We find that the Fröhlich scattering rate is directly related to the variance of the phonon fluctuations $\left\langle {\mathrm{q̅}}^{2}\right\rangle$ , as described within rate theories for time-dependent Peierls coupling,^63–65^ since scattering proceeds through the time-dependent coordinates *q*(*t*). To clearly see this, consider the multilayer dependence of the Fröhlich scattering rate *K*_*FS*_ which can be obtained by analyzing the time-dependent couplings *V*_*FS*_(*t*) = $\left\langle -,k\left|{Ĥ}_{bX}\left(t\right)\right|-,k^{\prime }\right\rangle$ ∝ $\left\langle -,k\left|P_{B}{Ĥ}_{bX}\left(t\right)P_{B}\right|-,k^{\prime }\right\rangle$ where $P_{-}$ is a lower polariton projection operator. For such a time-dependent coupling term *V*_*FS*_(*t*) the rate constant is known^63–65^ to be proportional to the variance $\left\langle V_{FS}^{2}\left(t\right)\right\rangle$ . Therefore, the Fröhlich scattering rate constant *K*_*FS*_ is directly proportional to $\left\langle {\mathrm{q̅}}^{2}\right\rangle$ (following the steps in eq 28 - eq 29) and is then expressed as

31 $$K_{FS}\left(N_{L}\right)=A_{FS}\cdot \frac{1}{S^{2}}\overset{N_{L}}{\underset{m}{\sum }}\sin^{4}\left(k_{0}\cdot y_{m}\right)$$

where *N*_*L*_ is the number of layers and *A* is a prefactor. The prefactor can also be written as *A*_*FS*_ = *K*_*FS*_(1) which corresponds to the *K*_*FS*_ in the single-layer material setup which we use in our analytical expression.

In Figure 5 we numerically analyze the Fröhlich scattering rate in the lower polariton for the data presented in Figure 2 and Figure 4. We compute the relative lower polariton population inside and outside the *k*-window ( k̅±5·(2π/Nα) with the initial excitation centered around k̅ ) within which the system was excited in the upper polariton. Figure 5a-b show that the relative inside population *P*_*in*_/*P*_–_ decays less significantly for 10 layers compared to a single layers. We fit this relative inside population (see Figure 5c) to an exponential curve (i.e., $a\cdot e^{-K_{FS}t}+b$ where *a* and *b* are scalar constants) to estimate the rate (*K*_*FS*_) of Fröhlich scattering induced by the phonons.

**Figure 5:**
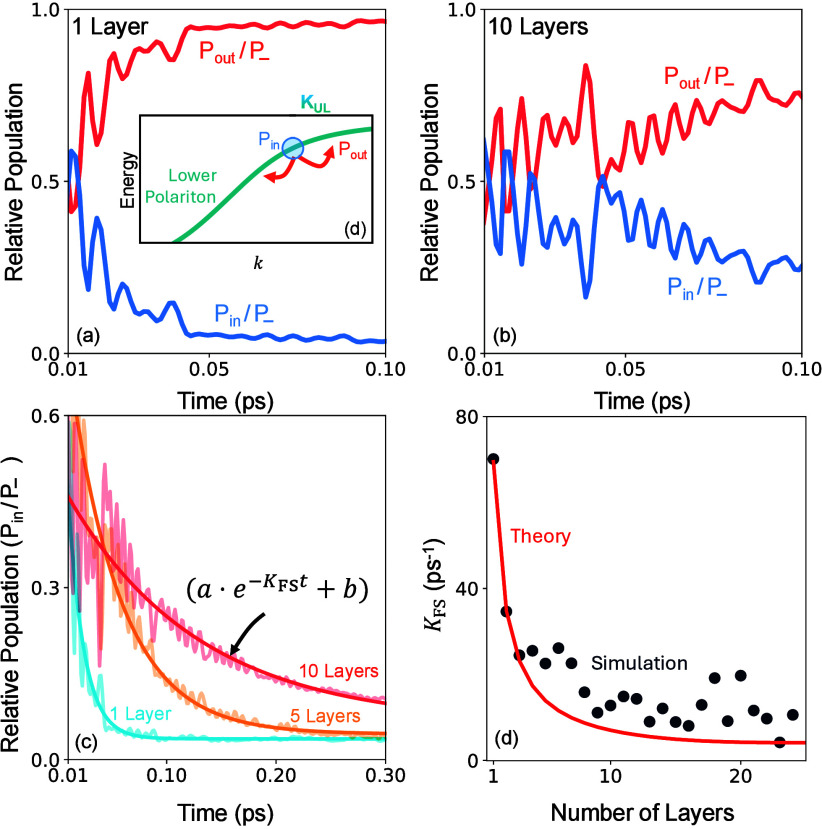
Population inside and outside the excitation window. (a and b) Population, relative to the lower polariton, inside the coherent relaxation window vs population scattering outside this window in a single layer and in 10 layers. (c) Inside population and fitted exponential function for 1, 5, and 10 layers. (d) Fitted *K*_*FS*_ compared to our analytical expression as a function of the number of layers.

Figure 5d presents the layer dependent Fröhlich scattering rate computed numerically compared to the analytical scaling expression provided in eq 31. Overall, our results demonstrate that the qualitative layer-dependence of Fröhlich scattering rate is very well captured by our theoretical expression validating the microscopic mechanisms revealed in this work. We emphasize that the key component to capturing this phonon fluctuation synchronization is the inclusion of multiple material layers in the light–matter Hamiltonian and consequently the delocalized nature of the exciton along the quantization direction.

## Polariton Relaxation Rates

Figure 6 presents the how population dynamics is modified when altering the thickness of the material (by altering the number of layers). Figure 6a-c show that increasing the number of layers drastically reduces the lower polariton population due to the competition from the dark states that are excitonic. We also observe this in the upper-to-lower polariton rate constant *K*_*UL*_ that decreases with increase in number of layers. Following the same strategy as in the case of Fröhlich scattering rate constant, we arrive at the following analytical expression

32 $$K_{UL}\left(N_{L}\right)=A_{UL}\cdot \frac{1}{S^{2}}\overset{N_{L}}{\underset{m}{\sum }}\sin^{4}\left(k_{0}\cdot y_{m}\right)$$

**Figure 6:**
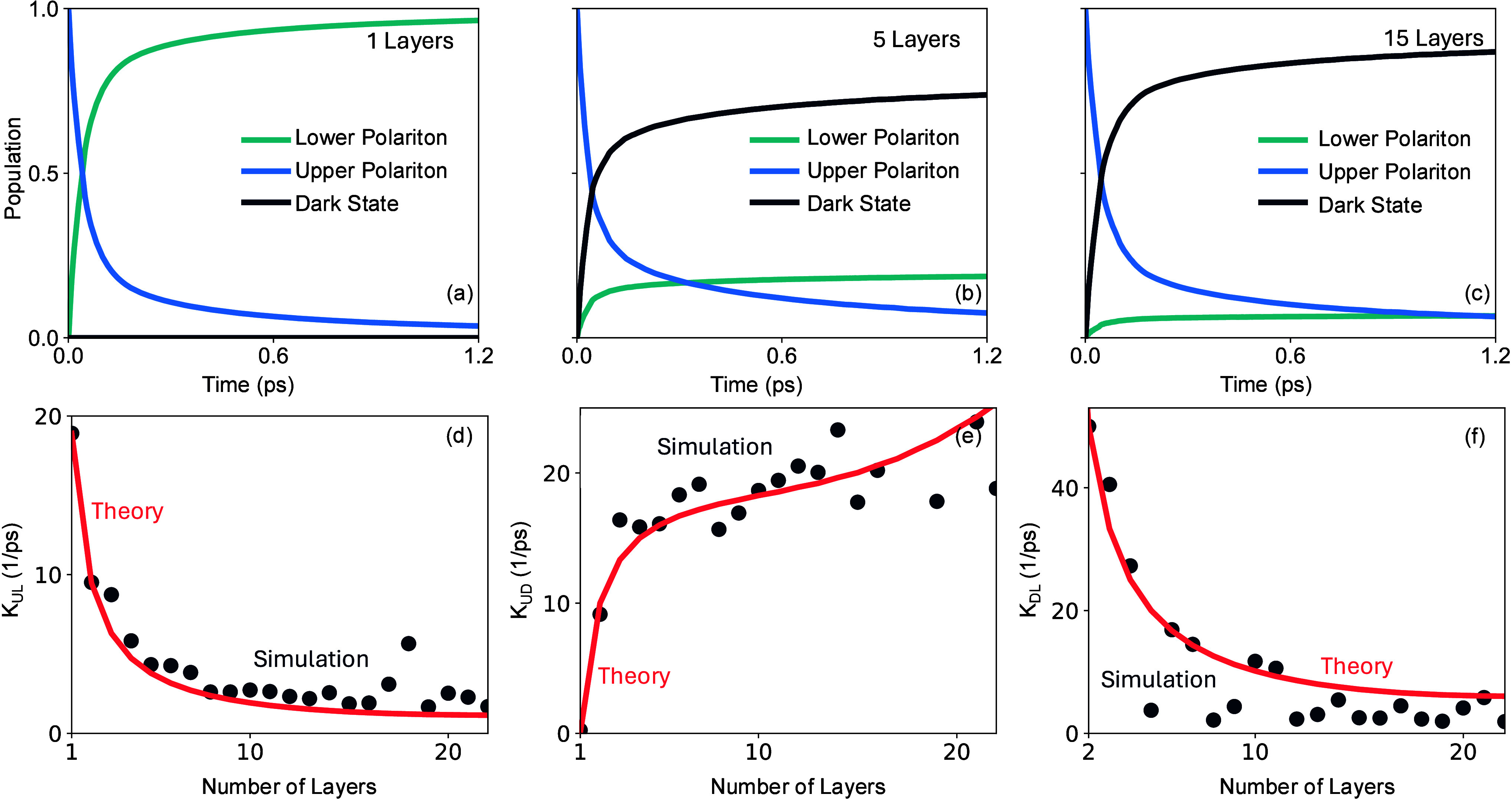
Effect of number of layers on polariton dynamics and kinetics. (a–c) Population dynamics for 1, 5, and 15 layers, initialized at 3.5 eV and using ω = 720 cm^–1^, τ = 400 cm^–1^. (d–f) Relaxation rate from upper polariton to lower polariton *K*_*UL*_ (d), upper to dark *K*_*UD*_ (e), and dark to lower *K*_*DL*_ (f), vs number of layers for excitation in the upper polariton at 3.5 eV.

Just as before we use *A*_*UL*_ = *K*_*UL*_(1) and obtain the theoretical curve presented in Figure 6d. Our analytical theory accurately captures the numerically extracted upper-to-lower rate constant.

The same strategy can again be utilized to provide an analytical expression for the upper-to-dark relaxation rate constant. Here, the relevant time-dependent coupling term between the upper polariton to the *d*th dark layer is $V_{UD}^{d}\left(t\right)=\left\langle +,k\left|{Ĥ}_{bX}\left(t\right)\right|k,d\right\rangle$ where $\left|k,d\right\rangle ={\mathrm{X̂}}_{k,d}^{†}\left|\mathrm{0̅}\right\rangle$ . Using this, we find that the overall upper-to-dark relaxation rate constant (which sums over all dark layers) can be written as

33 $$K_{UD}\left(N_{L}\right)=A_{UD}\underset{m}{\sum }\frac{\sin^{2}\left(k_{0}\cdot y_{m}\right)}{S}\underset{d}{\sum }D_{m,d}^{2}\approx A_{UD}\left(N_{L}-1\right)\cdot \underset{m}{\sum }\frac{\sin^{4}\left(k_{0}\cdot y_{m}\right)}{S^{2}}$$

where *A*_*UD*_ is a prefactor (which we obtain via fitting), and we have made the approximate replacement $\underset{d}{\sum }D_{m,d}^{2}\approx \left(N_{L}-1\right)\sin^{2}\left(k_{0}\cdot y_{m}\right)/S$ to provide a simple analytical expression. As a result of the additional (*N*_*L*_ – 1), the upper-to-dark polariton relaxation rate constant *K*_*UD*_ rapidly increases with the number of layers and then saturates at a certain number of layers. Note that the late rise in the rate constant (see red solid line in Figure 6e) after ∼ 15 layers found in our theory vanishes when not making the approximation in the second line in eq 33.

Similarly, the dark-to–lower polariton relaxation rate can be described within the same analytical framework. The relevant time-dependent coupling between the lower polariton and the *d*-th dark layer is given by $V_{DL}^{d}\left(t\right)=\left\langle k,d\left|{Ĥ}_{bX}\left(t\right)\right|-,k\right\rangle$ , where $\left|k,d\right\rangle ={\mathrm{X̂}}_{k,d}^{†}\left|\mathrm{0̅}\right\rangle$ . Using this coupling, the total dark-to–lower polariton relaxation rate constant can be expressed as follows

34 $$K_{DL}\left(N_{L}\right)=A_{DL}\underset{m}{\sum }\frac{\sin^{2}\left(k_{0}\cdot y_{m}\right)}{S}\underset{d}{\sum }D_{m,d}^{2}\approx A_{DL}\underset{m}{\sum }\frac{\sin^{4}\left(k_{0}\cdot y_{m}\right)}{S^{2}}$$

Here, *A*_*DL*_ = *K*_*DL*_(1), from which we obtain the theoretical curve shown in Figure 6f. The analytical model accurately reproduces the numerically extracted dark-to-lower rate constant. The factor *N*_*L*_ – 1 does not appear because the rate equation $\overset{N-1}{\underset{d}{\sum }}\frac{dP_{d}\left(t\right)}{dt}\approx -\overset{N-1}{\underset{d}{\sum }}K_{DL}P_{d}\left(t\right)$ is summed over *d* on both sides. Note that while the lower polariton can also be populated via upper-to-dark and then dark-to-lower polariton relaxation, such a two-step incoherent/rate-like process plays a relatively insignificant role when compared to a direct upper-to-lower polariton relaxation process for the range of time scales (picoseconds) presented in this work. Overall, the phonon fluctuation synchronization effect successfully explains various relaxation processes, specifically how they are modified in a material of finite thickness placed inside an optical cavity.

In conclusion, we have explored exciton-polariton relaxation dynamics following an excitation in the upper polariton branch. Using direct mixed quantum-classical quantum dynamical simulations as well as analytical analysis, we provide the mechanistic principles that govern exciton-polariton relaxation and show how the finite thickness of materials modifies these processes.

Our results demonstrate that the polariton relaxation proceeds through a two-step mechanism involving an interband vertical transition followed by intraband phonon-mediated Fröhlich scattering. We provide an analytical criterion for the molecular lattice constant; typical molecular parameters satisfy this criterion, enabling such vertical transitions.

Furthermore, we find that the second step, namely the Fröhlich scattering within the lower polariton band, is significantly suppressed when the finite thickness of a material is taken into consideration. We attribute this reduction to the phonon-fluctuation synchronization effect, where the spatial delocalization of polaritons induces self-averaging of phonon fluctuations across layers along the quantization axis, effectively weakening Fröhlich scattering in the lower polariton. As a result, cavities hosting materials of finite thickness (i.e., multilayered materials) exhibit a long-lived, *k*-localized polaritonic density upon polariton relaxation. We show that an analytical description of various relaxation processes can be developed based on the phonon-fluctuation self-averaging mechanism. We find that increasing the number of layers suppresses both upper-to-lower and dark-to-lower polariton relaxation, whereas upper-to-dark polariton relaxation initially increases and then saturates beyond a certain number of layers. We find that our analytical description accurately captures our numerical results.

We anticipate that the proposed mechanisms could be verified experimentally. First, the suppression of the intraband Fröhlich scattering in multilayered materials can be verified by comparing the time-resolved, angle-resolved photoluminescence spectra by exciting in the upper polariton near the anticrossing point (where both polaritons are 50% excitonic and 50% photonic) at two different material thicknesses. Our prediction is that, (at strong coupling regime) at higher thickness, the material will exhibit lower Fröhlich scattering in the lower polariton. Second, experimental platforms where coherent phonons can be launched would be able to deliberately move away from the uncorrelated phonon picture and mimic the dynamics presented in Figure 4e-f. We anticipate that our present work will inspire such experiments that reveals the mechanistic principles of exciton-polariton relaxation.

Finally, we also note that while we expect our results to be generic, our present work uses a parametrized model Hamiltonian which do not incorporate the nonlinear exciton–phonon coupling, anharmonic effects of molecular vibrations, the nonlinear many-body effects,^36^ and treat the phonons quasi-classically. We plan to address these deficiencies in the future via a direct *ab initio* many-body simulation of exciton-polaritons or pursuing a more accurate (mesoscale) quantum dynamical approach beyond the mixed quantum-classical treatment.^66^ Overall, our work identifies the key pathways of exciton-polariton relaxation and develops a general mechanistic and predictive theory for relaxation in realistic filled cavities or cavities coupled to material of finite thickness.

## Supplementary Material



## Data Availability

The data that support the plots within this paper and other findings of this study are available from the corresponding authors upon a reasonable request. We developed our code based on our semiclassical quantum dynamics code available at https://github.com/mandalgrouptamu/SQD. The source code that supports the findings of this study are available from the corresponding author upon reasonable request.

## Notes

Justification of layer-scaling approximation; temperature dependence of transition matrix (PDF)

## References

[1] Hutchison J. A., Schwartz T., Genet C., Devaux E., Ebbesen T. W. (2012). Modifying
chemical landscapes by coupling to vacuum fields. Angew. Chem., Int. Ed..

[2] Basov D. N., Asenjo-Garcia A., Schuck P. J., Zhu X., Rubio A. (2020). Polariton
panorama. Nanophotonics.

[3] Mandal A., Taylor M. A., Weight B. M., Koessler E. R., Li X., Huo P. (2023). Theoretical advances
in polariton chemistry and molecular cavity
quantum electrodynamics. Chem. Rev..

[4] Li T. E., Cui B., Subotnik J. E., Nitzan A. (2022). Molecular polaritonics: Chemical
dynamics under strong light–matter coupling. Annu. Rev. Phys. Chem..

[5] Hu W., Gustin I., Krauss T. D., Franco I. (2022). Tuning and enhancing
quantum coherence time scales in molecules via light-matter hybridization. J. Phys. Chem. Lett..

[6] Foley J. J., McTague J. F., DePrince A. E. (2023). Ab initio methods
for polariton chemistry. Chemical Physics Reviews.

[7] Opala A., Matuszewski M. (2023). Harnessing
exciton-polaritons for digital computing,
neuromorphic computing, and optimization. Optical
Materials Express.

[8] Sanvitto D., Kéna-Cohen S. (2016). The road towards polaritonic devices. Nat. Mater..

[9] Zasedatelev A. V., Baranikov A. V., Urbonas D., Scafirimuto F., Scherf U., Stöferle T., Mahrt R. F., Lagoudakis P. G. (2019). A room-temperature
organic polariton transistor. Nat. Photonics.

[10] Amo A., Liew T., Adrados C., Houdré R., Giacobino E., Kavokin A., Bramati A. (2010). Exciton–polariton
spin switches. Nat. Photonics.

[11] Ballarini D., Gianfrate A., Panico R., Opala A., Ghosh S., Dominici L., Ardizzone V., De Giorgi M., Lerario G., Gigli G. (2020). others Polaritonic neuromorphic
computing outperforms linear classifiers. Nano
Lett..

[12] Ghosh S., Nakajima K., Krisnanda T., Fujii K., Liew T. C. (2021). Quantum
neuromorphic computing with reservoir computing networks. Advanced Quantum Technologies.

[13] Mirek R., Opala A., Comaron P., Furman M., Król M., Tyszka K., Seredyński B., Ballarini D., Sanvitto D., Liew T. C. (2021). others Neuromorphic
binarized polariton networks. Nano Lett..

[14] Rojas-Sánchez J. A., Jomaso Y. A. G., Vargas B., Domínguez D. L., Ordoñez-Romero C. L., Lara-García H. A., Camacho-Guardian A., Pirruccio G. (2023). Topological Frenkel exciton polaritons
in one-dimensional lattices of strongly coupled cavities. Phys. Rev. B.

[15] Ghosh S., Liew T. C. (2020). Quantum computing with exciton-polariton condensates. npj Quantum Information.

[16] Berloff N. G., Silva M., Kalinin K., Askitopoulos A., Töpfer J. D., Cilibrizzi P., Langbein W., Lagoudakis P. G. (2017). Realizing
the classical XY Hamiltonian in polariton simulators. Nat. Mater..

[17] Xiang B., Xiong W. (2024). Molecular polaritons for chemistry, photonics and quantum technologies. Chem. Rev..

[18] Nagarajan K., Thomas A., Ebbesen T. W. (2021). Chemistry under
vibrational strong
coupling. J. Am. Chem. Soc..

[19] Thomas A., Lethuillier-Karl L., Nagarajan K., Vergauwe R. M., George J., Chervy T., Shalabney A., Devaux E., Genet C., Moran J. (2019). others Tilting a ground-state reactivity landscape
by vibrational strong coupling. Science.

[20] Ahn W., Triana J. F., Recabal F., Herrera F., Simpkins B. S. (2023). Modification
of ground-state chemical reactivity via light–matter coherence
in infrared cavities. Science.

[21] Bhuyan R., Mony J., Kotov O., Castellanos G. W., Gómez Rivas J., Shegai T. O., Borjesson K. (2023). The rise and
current status of polaritonic photochemistry and photophysics. Chem. Rev..

[22] Rashidi K., Michail E., Salcido-Santacruz B., Paudel Y., Menon V. M., Sfeir M. Y. (2025). Efficient and tunable
photochemical charge transfer
via long-lived bloch surface wave polaritons. Nat. Nanotechnol..

[23] Li T. E., Tao Z., Hammes-Schiffer S. (2022). Semiclassical
real-time nuclear-electronic
orbital dynamics for molecular polaritons: Unified theory of electronic
and vibrational strong couplings. J. Chem. Theory
Comput..

[24] Yu Q., Hammes-Schiffer S. (2022). Multidimensional
quantum dynamical simulation of infrared
spectra under polaritonic vibrational strong coupling. J. Phys. Chem. Lett..

[25] Li T. E., Hammes-Schiffer S. (2023). QM/MM modeling of vibrational polariton
induced energy
transfer and chemical dynamics. J. Am. Chem.
Soc..

[26] Brawley Z. T., Pannir-Sivajothi S., Yim J. E., Poh Y. R., Yuen-Zhou J., Sheldon M. (2025). Vibrational weak and strong coupling modify a chemical
reaction via cavity-mediated radiative energy transfer. Nat. Chem..

[27] Manjalingal A., Rahmanian Koshkaki S., Blackham L., Mandal A. (2025). Tilted Material
in
an Optical Cavity: Light-Matter Moiré Effect and Coherent Frequency
Conversion. ACS Photonics.

[28] Yang Z., Bhakta H. H., Xiong W. (2023). Enabling multiple intercavity
polariton
coherences by adding quantum confinement to cavity molecular polaritons. Proc. Natl. Acad. Sci. U. S. A..

[29] Tichauer R. H., Feist J., Groenhof G. (2021). Multi-scale
dynamics simulations
of molecular polaritons: The effect of multiple cavity modes on polariton
relaxation. J. Chem. Phys..

[30] Xu D., Mandal A., Baxter J. M., Cheng S.-W., Lee I., Su H., Liu S., Reichman D. R., Delor M. (2023). Ultrafast imaging of
polariton propagation and interactions. Nat.
Commun..

[31] Chng B. X., Mondal M. E., Ying W., Huo P. (2025). Quantum dynamics simulations
of exciton polariton transport. Nano Lett..

[32] Ying W., Chng B. X., Delor M., Huo P. (2025). Microscopic theory
of polariton group velocity renormalization. Nat. Commun..

[33] Krupp N., Groenhof G., Vendrell O. (2025). Quantum dynamics
simulation of exciton-polariton
transport. Nat. Commun..

[34] Sokolovskii I., Luo Y., Groenhof G. (2025). Disentangling enhanced
diffusion and ballistic motion
of excitons coupled to Bloch surface waves with molecular dynamics
simulations. J. Phys. Chem. Lett..

[35] Blackham L., Manjalingal A., Koshkaki S. R., Mandal A. (2025). Microscopic theory
of polaron-polariton dispersion and propagation. Nano Lett..

[36] Ghosh P., Manjalingal A., Wickramasinghe S., Koshkaki S. R., Mandal A. (2025). Mean-field
mixed quantum-classical approach for many-body quantum dynamics of
exciton polaritons. Phys. Rev. B.

[37] Pérez-Sánchez J. B., Yuen-Zhou J. (2025). Radiative pumping vs vibrational relaxation of molecular
polaritons: a bosonic mapping approach. Nat.
Commun..

[38] Wang S., Hsu L.-Y., Chen H.-T. (2025). Robust
Surface-Induced Enhancement
of Exciton Transport in Magic-Angle-Oriented Molecular Aggregates. J. Phys. Chem. Lett..

[39] Catuto R. F., Chen H.-T. (2025). Interplay between static and dynamic
disorder: Contrasting
effects on dark state population inside a cavity. J. Chem. Phys..

[40] Aroeira G. J. R., Ribeiro R. F. (2025). Static disorder-induced
renormalization of polariton
group velocity. J. Chem. Phys..

[41] Neuman T., Aizpurua J. (2018). Origin of the asymmetric
light emission from molecular
exciton–polaritons. Optica.

[42] Groenhof G., Climent C., Feist J., Morozov D., Toppari J. J. (2019). Tracking
polariton relaxation with multiscale molecular dynamics simulations. journal of physical chemistry letters.

[43] Sokolovskii I., Blumberger J. (2025). Strong intermolecular
coupling protects delocalization
and transport of organic exciton-polaritons against static excitation
energy disorder. J. Chem. Phys..

[44] Pandya R., Ashoka A., Georgiou K., Sung J., Jayaprakash R., Renken S., Gai L., Shen Z., Rao A., Musser A. J. (2022). Tuning the Coherent
Propagation of Organic Exciton-Polaritons
through Dark State Delocalization. Advanced
Science.

[45] Mandal A., Xu D., Mahajan A., Lee J., Delor M., Reichman D. R. (2023). Microscopic
theory of multimode polariton dispersion in multilayered materials. Nano Lett..

[46] Hong Y., Xu D., Delor M. (2026). Exciton delocalization
suppresses polariton scattering. Chem..

[47] Polak D., Jayaprakash R., Lyons T. P., Martínez-Martínez L. A., Leventis A., Fallon K. J., Coulthard H., Bossanyi D. G., Georgiou K., Petty A. J. (2020). others
Manipulating molecules with strong coupling: harvesting triplet excitons
in organic exciton microcavities. Chemical science.

[48] Pandya R., Ashoka A., Georgiou K., Sung J., Jayaprakash R., Renken S., Gai L., Shen Z., Rao A., Musser A. J. (2022). Tuning the coherent
propagation of organic exciton-polaritons
through dark state delocalization. Advanced
Science.

[49] Alam S., Liu Y., Holmes R. J., Frontiera R. R. (2025). Quantification of Nuclear Coordinate
Activation on Polaritonic Potential Energy Surfaces. arXiv.

[50] Lydick N., Hu J., Deng H. (2024). Dimensional
dependence of a molecular-polariton mode
number. J. Opt. Soc. Am. B.

[51] Sun K., Du M., Yuen-Zhou J. (2025). Exploring
the Delocalization of Dark States in a Multimode
Optical Cavity. J. Phys. Chem. C.

[52] Hoffmann N. M., Schäfer C., Rubio A., Kelly A., Appel H. (2019). Capturing
vacuum fluctuations and photon correlations in cavity quantum electrodynamics
with multitrajectory Ehrenfest dynamics. Phys.
Rev. A.

[53] Luk H. L., Feist J., Toppari J. J., Groenhof G. (2017). Multiscale molecular
dynamics simulations of polaritonic chemistry. J. Chem. Theory Comput..

[54] Koshkaki S. R., Manjalingal A., Blackham L., Mandal A. (2025). Exciton-Polariton
Dynamics
in Multilayered Materials. arXiv.

[55] Keeling J., Kéna-Cohen S. (2020). Bose–Einstein condensation
of exciton-polaritons
in organic microcavities. Annu. Rev. Phys. Chem..

[56] Hoffmann N. M., Lacombe L., Rubio A., Maitra N. T. (2020). Effect of many modes
on self-polarization and photochemical suppression in cavities. J. Chem. Phys..

[57] Li X., Tully J. C., Schlegel H. B., Frisch M. J. (2005). Ab initio Ehrenfest
dynamics. J. Chem. Phys..

[58] Janke S. M., Qarai M. B., Blum V., Spano F. C. (2020). Frenkel–Holstein
Hamiltonian applied to absorption spectra of quaterthiophene-based
2D hybrid organic–inorganic perovskites. J. Chem. Phys..

[59] Mandal A., Krauss T. D., Huo P. (2020). Polariton-mediated
electron transfer
via cavity quantum electrodynamics. J. Phys.
Chem. B.

[60] Koessler E. R., Mandal A., Musser A. J., Krauss T. D., Huo P. (2025). Polariton
mediated electron transfer under the collective molecule–cavity
coupling regime. Chem. Sci..

[61] Chowdhury S. N., Mandal A., Huo P. (2021). Ring polymer
quantization of the
photon field in polariton chemistry. J. Chem.
Phys..

[62] Wickramasinghe, S. ; Amini, A. A. ; Mandal, A. On-the-fly Cavity–Molecular Dynamics of Vibrational Polaritons. Phys. Chem. Chem. Phys. 2026. 10.1039/D5CP04879F 41837315

[63] Chowdhury S. N., Mandal A., Huo P. (2021). Ring polymer quantization of the
photon field in polariton chemistry. J. Chem.
Phys..

[64] Troisi A., Nitzan A., Ratner M. A. (2003). A rate
constant expression for charge
transfer through fluctuating bridges. J. Chem.
Phys..

[65] Beratan D.
N., Skourtis S. S., Balabin I. A., Balaeff A., Keinan S., Venkatramani R., Xiao D. (2009). Steering electrons on moving pathways. Accounts
of chemical research.

[66] Citty B., Lynd J. K., Gera T., Varvelo L., Raccah D. I. (2024). MesoHOPS:
Size-invariant scaling calculations of multi-excitation open quantum
systems. J. Chem. Phys..

