# Supplementary material for: Mechanistic Principles of Exciton–Polariton Relaxation
Source: J Phys Chem Lett. 2026 Apr 3;17(15):4535–45. doi: 10.1021/acs.jpclett.6c00405 (PMC13093670; doi:10.1021/acs.jpclett.6c00405)
Supplement: Supplementary file 1 [file jz6c00405_si_001.pdf]

Mechanistic Principles of Exciton–Polariton Relaxation

Ian Haines†, Arshath Manjalingal†, Logan Blackham,
Saeed Rahmanian Koshkaki*, Arkajit Mandal*

March 3, 2026

Contents

S1 Justification of the layer-scaling approximation **2**

S2 Temperature dependence of Transition Matrix **3**

*Corresponding Authors: rahmanian@tamu.edu, mandal@tamu, † Equal contribution

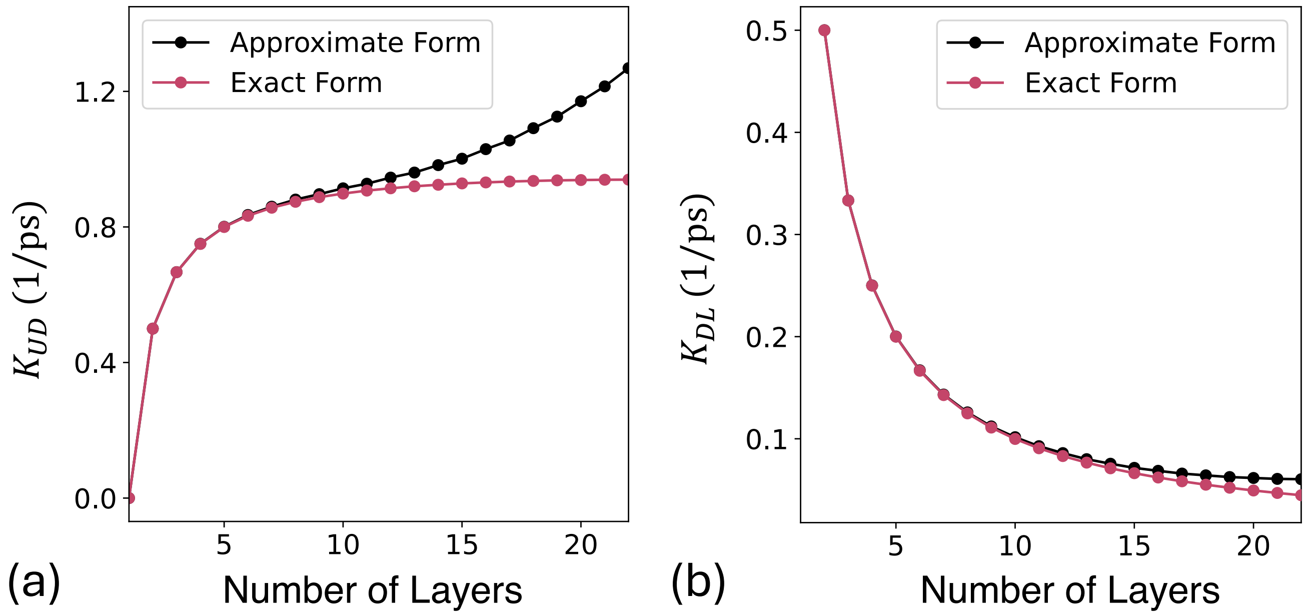

Figure S1: Exact analytical (red) and approximate analytical (black) relaxation rates as a function of the number of layers. (a) K_{UD} exhibits an $(N_L - 1)$ scaling and saturates at large N_L. (b) K_{DL} shows weak layer dependence and is well captured by the analytical approximation. Here, the constants A_{UD} and A_{DL} are set to one.

S1 Justification of the layer-scaling approximation

As it was shown in the main text the analytical expression for the relaxation from the upper polariton to dark states can be written as

$$K_{UD}(N_L) = A_{UD} \sum_m \frac{\sin^2(k_0 \cdot y_m)}{S} \sum_d \mathcal{D}^2_{m,d}. \tag{S1}$$

We approximate the dark layer overlap term as

$$\sum_d \mathcal{D}^2_{m,d} \approx (N_L - 1) \frac{\sin^2(k_0 \cdot y_m)}{S}. \tag{S2}$$

Where N_L is the number of layers. Using this approximation, Eq. (S1) can be rewritten as

$$K_{UD} \approx A_{UD}(N_L - 1) \sum_m \frac{\sin^4(k_0 \cdot y_m)}{S^2}. \tag{S3}$$

This approximation follows from the observation that the dark-layer coefficients ($\mathcal{D}_{m,d}$) are nearly identical across layers. In Fig. S1(a), we compare the exact analytical expression in Eq. (S1) with the approximate form in Eq. (S3), and find good agreement especially for a lesser number layers (up to 10 layers for $\alpha_y = 40$ Å).

Similar to the relaxation from upper to dark, the relaxation rate from all the dark states to the lower polariton can be written as

$$\begin{aligned} K_{DL}(N_L) &= A_{DL} \sum_m \frac{\sin^2(k_0 \cdot y_m)}{S} \sum_d \mathcal{D}^2_{m,d} \\ &\approx A_{DL} \sum_m \frac{\sin^4(k_0 \cdot y_m)}{S^2}. \end{aligned} \tag{S4}$$

As shown in Fig. S1(b), the exact and approximate analytical expressions for K_{DL} are in good agreement. Since the $1/N_L - 1$ term cancels in the approximate equation, relaxation is allowed when there is one layer despite the dark states not existing for a single layer.

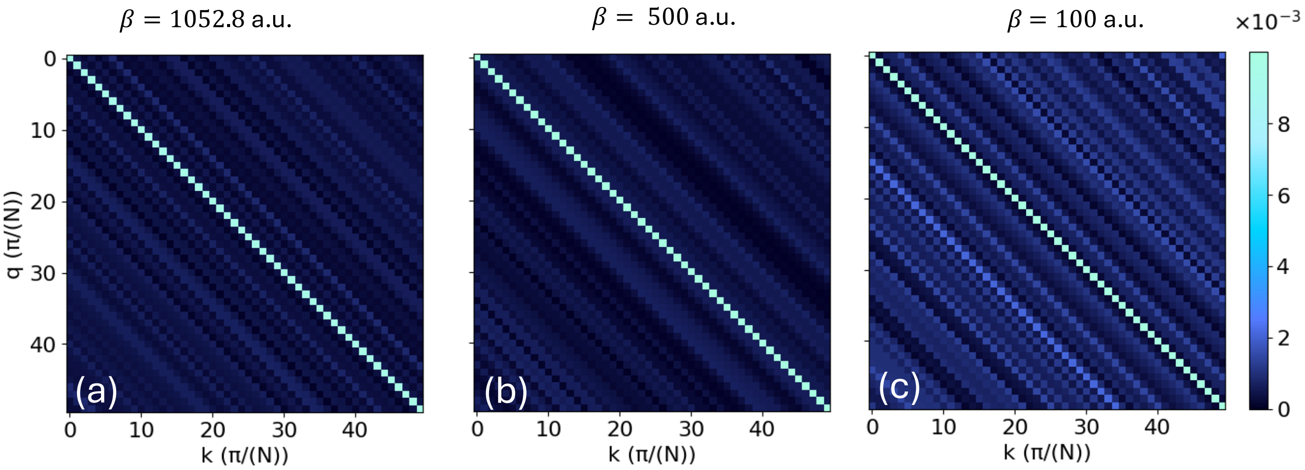

Figure S2: Transition matrices illustrating $k - k'$ transitions at different inverse temperatures: (a) $\beta = 1052.8$ a.u. (300 k) (b) $\beta = 500$ a.u. (c) $\beta = 100$ a.u.

S2 Temperature dependence of Transition Matrix

Fig. S2 shows that the broadening of the vertical transition increases with temperature. Here, we include the first and second terms of Eq. (24) from the main text. As seen clearly in Fig. S2(c), the off-diagonal terms become more dominant.
